# The Impact of Reoperations Following Bariatric Surgery on Mid-term Outcomes

**DOI:** 10.1007/s11695-023-06519-0

**Published:** 2023-02-24

**Authors:** Jennifer Straatman, Ahmet Demirkiran, Niels J. Harlaar, Huib A. Cense, Dutch Audit for Treatment of Obesity Group (DATO), Frederik H. W. Jonker

**Affiliations:** 1 Department of Gastrointestinal Surgery, Rode Kruis Ziekenhuis, Vondellaan 13, 1942 LE, Beverwijk, Netherlands; 2 Dutch Institute for Clinical Auditing, Leiden, Netherlands

**Keywords:** Mid-term follow-up, Reoperations, Percentage total weight loss, Comorbidities

## Abstract

**Purpose:**

With the obesity epidemic, the number of bariatric procedures is increasing, and although considered relatively safe, major postoperative complications still occur. In cancer surgery, major complications such as reoperations have been associated with deteriorated mid/long-term outcomes. In obesity surgery, the effects of reoperations on postoperative weight loss and associated comorbidities remain unclear. The aim of this study was to assess mid-term weight loss and comorbidities following early reoperations in obesity surgery.

**Methods:**

A population-based cohort study was performed within the Dutch Audit for Treatment of Obesity (DATO), including all patients that underwent a primary gastric bypass procedure or sleeve gastrectomy. Follow-up data was collected up until 5 years postoperatively on percentage total weight loss (%TWL) and comorbidities.

**Results:**

A total of 40,640 patients underwent a gastric bypass procedure or sleeve gastrectomy between 2015 and 2018. Within this cohort, 709 patients (1.7%) suffered a major complication requiring reoperation within 30 days. %TWL at 24 months was 33.1 ± 9.2 in the overall population, versus 32.9 ± 8.7 in the patients who underwent a reoperation (*p*=0.813). Both analysis per year and Cox regression techniques revealed no differences in long-term follow-up regarding percentage TLW, and weight loss success rates (%TWL>20%) in patients who underwent a reoperation compared to patients without reoperation. At 5 years, the availability of follow-up data was low. No differences were observed in the remission of comorbidities.

**Discussion:**

Major complications requiring reoperation within 30 days of gastric bypass surgery or sleeve gastrectomy did not affect long-term outcomes with regard to weight loss or remission of comorbidities.

**Graphical Abstract:**

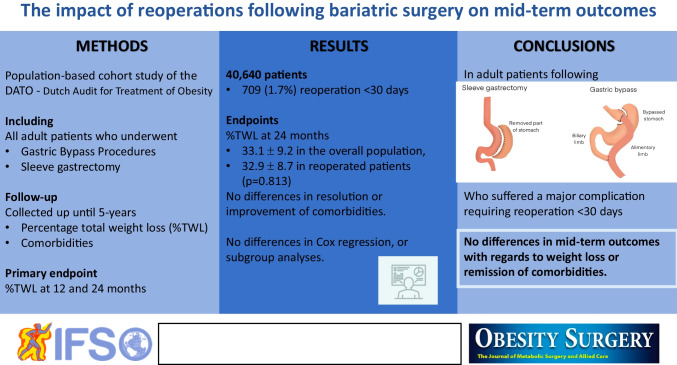

## Introduction

Although the safety of bariatric surgery has improved over the past decades, with the adoption of minimally invasive techniques, centralization, standardized peri-operative care protocols, and the implementation of accreditation programs [1], a severe complicated postoperative course is still observed in 2.87% of cases [2]. Whilst the number of bariatric surgeries performed increases annually with the obesity epidemic, the mid-to-long-term effects of these major complications, such as reoperations, remain unclear [3, 4].

With postoperative complications affecting long-term outcomes following gastrectomy with Roux and Y reconstruction for cancer [5], one could question whether postoperative complications following bariatric surgery affect long-term outcomes as well. It should be noted that patients undergoing gastric surgery for the treatment of morbid obesity are completely different from oncologic patients operated on for gastric cancer. However, adherence to a healthy diet and adequate physical exercise may be hindered by major early complications after bariatric surgery. In addition, the patient’s motivation may also be affected by a complicated postoperative course, potentially leading to inferior outcomes. Furthermore, prolonged inflammation could lead to micronutrient deficiencies in these patients, as described previously in colorectal cancer patients [6].

Data on long-term follow-up after bariatric surgery often focusses on weight loss success and the need for revisional surgery, with studies showing that up to 27.8% of patients require revisional surgery during follow-up [7]. The effect of major complications following primary surgery, such as reoperations within 30 days of index surgery, on mid-to-long-term outcomes, was not discussed in larger follow-up cohort series [8, 9]. Only two small studies reported on the effects of complications on weight loss up until 12 months after surgery. Both studies found no differences in weight loss or incidence of comorbidities in the follow-up after sleeve gastrectomy or gastric bypass [10, 11].

Here, we assessed the long-term effects of a reoperation within 30 days of primary bariatric surgery on total weight loss and remission of co-morbidities.

## Methods

All procedures performed in this study were in accordance with the ethical standards of the institutional and/or national research committee and with the 1964 Helsinki Declaration and its later amendments or comparable ethical standards [12].

### Source of Data

A population-based cohort study was performed within the Dutch Audit for Treatment of Obesity (DATO). In the Netherlands, bariatric surgery is centralized and performed only in hospitals with a case volume of more than 200 procedures annually. Hospitals are mandated to collect data for the DATO registry, allowing for quality control and research [2]. Research protocols are assessed for study design, statistical plan, and ethical considerations in the DATO meetings.

### Participants

All patients who underwent bariatric surgery between January 2015 and December 2018 in the Netherlands were included in this study. A minimal follow-up had to be available for at least 30 days postoperatively. Included procedures entailed gastric bypass and sleeve gastrectomy. Gastric bypass surgery included Roux and Y gastric bypass, single anastomosis gastric bypass, and banded gastric bypass procedures. For sleeve gastrectomy, stapling only and buttressing techniques were allowed. For comparability, only minimally invasive procedures were included. Revisional procedures were not included in this study.

### Outcome

The primary outcome was percentage total weight loss (%TWL) at 12 and 24 months, compared between patients who underwent a reoperation versus patients with no reoperations following bariatric surgery. TWL was calculated as the weight loss (or gain) in kilograms, divided by baseline weight. Early reoperations encompassed all surgical procedures under general anesthesia, within 30 days of the index operation. Secondary outcomes included incidence of comorbidities and TWL over time (Cox-regression), again compared for patients following a reoperation versus those without reoperations following bariatric surgery. Alongside the TWL success rate reported, a bariatric procedure is considered successful if the percentage of total weight loss is over 20% [13]. Secondary outcomes included remission or improvement of pre-existent co-morbidities during follow-up after bariatric surgery. Remission or improvement of comorbidities was defined as either a decreased need (or no need) for therapy of comorbidities, resulting in either decreased intake or dosage of medication, or no longer requiring interventions such as obstructive sleep apnea mask.

### Collected Data

Assessment of comorbidities included IFSO (International Federation for the Surgery of Obesity) criteria, which assess the presence of; hypertension defined as systole > 140 mmHg or diastole above 90 mmHg on repeated measurements. Diabetes is defined as a Hba1c outside normal ranges (20–42 mmol/mol). Dyslipidemia is defined as disturbances in the lipid spectrum (HDL, LDL, triglycerides), treated with medication. Gastro-esophageal reflux disease (GERD) is confirmed on gastroscopy and/or 24-h acid testing. Obstructive sleep apnea syndrome (OSAS) was confirmed on polysomnogram with apnea-hypnea index > 5. Joint pain encompassed patients diagnosed with a form of arthrosis receiving treatment from a medical specialist.

### Sample Size

In 2019, the rate of reinterventions was 1.7% [14], and the rate for reoperations following gastric bypass surgery in a large American database was 2.6% [15]. In order to assess long-term outcomes, and to correct for 14 different baseline characteristics (gender, age, BMI, IFSO co-morbidities, and other comorbidities), correction will be applied for 15 factors. Aiming for at least ten events per corrected variable, and the lowest reported incidence of 1.7%, this would entail 150 patients who underwent a reoperation, and thus a total dataset with at least 8824 patients. In the DATO registry, 40,640 patients underwent a primary bariatric procedure between January 2015 and December 2018. The dataset is therefore deemed to have a large enough sample size for the calculation of a prediction model with adequate power.

### Missing Data

We assumed missing data for baseline characteristics occurred at random. For the presented operative data, no data was missing regarding the type of surgery, access type, and type of gastric bypass procedure. The 39 patients who died within 30 days were excluded from further analysis; no differences in baseline characteristics were observed for these patients compared to patients who survived the past 30 days postoperatively. For baseline characteristics, per variable, less than 1% of data was missing. Using multiple imputation techniques taking into account all predictors, 20 new datasets were created with identical known information, but differences in imputed data to correct for uncertainty in imputed data. Pooled data from the 20 imputed datasets was used to correct the outcomes on long-term weight loss for baseline characteristics.

Follow-up data is most likely not missing at random, and therefore, imputation was not deemed appropriate. Hence, only the follow-up was analyzed for years 1 and 2, when more than 50% of data was available.

### Statistical Analysis

Analyses were performed using IBM SPSS statistics, version 26.0, Armonk, NY. Baseline characteristics and peri-operative data were assessed and compared for patients who underwent a reoperation within 30 days from the index operation compared to patients without early reoperations. Continuous variables were assessed for normal distribution and presented as mean and standard deviation for normal distributions and median and interquartile ranges (IQR) for non-normal distributed data. Continuous data was compared using a Student’s *T*-test and Mann-Whitney-*U* as appropriate. For nominal data, numbers are presented with frequency percentages and compared using chi-square tests. Bonferroni correction and standardized residuals were used to assess for differences between multiple groups.

Kaplan-Meier curves and subsequent Cox regression were used to longitudinally assess the percentage of successful weight loss in patients who underwent a reoperation compared to patients that did not require reoperations. The correction was applied for age, gender, baseline BMI, and comorbidities. Subgroup analyses were performed for patients who underwent a sleeve gastrectomy and patients who underwent a gastric bypass procedure.

## Results

A total of 40,640 patients underwent a laparoscopic bariatric procedure between 01-01-2015 and 31-12-2018, Baseline characteristics are reported in Table 1. Data is presented for all patients, and for the subgroup of patients who underwent a reoperation because of complications. All variables were recorded for 40,640 patients and assessment of missing data revealed no pattern, with less than 1% of values missing for each variable.

**Table 1:** Baseline characteristics

Parameter	Total population		Reoperation		*p*-value
Total no	40,640		709	1.7%	
Sex (male, %)	8507	20.9%	182	25.7%	0.002
Age (mean, SD)	44 ± 11.6		47 ± 10.7		<0.001
Length (mean, SD)	169.6 ± 8.9		169.8 ± 8.9		0.581
Weight (mean, SD)	126.1 ± 21.5		124.1 ± 19.6		0.031
BMI	43.7 ± 5.9		43.1 ± 5.5		0.015
ASA classification					
ASA 1	919	2.3%	14	2.0%	0.484*
ASA 2	21,108	51.9%	352	49.6%	
ASA 3	18,266	44.9%	227	32.0%	
ASA 4	183	0.5%	5	0.7%	
Screening morbidities	Present	%	Present	%	
Hypertension	9660	23.8%	205	28.9%	0.001
Diabetes	5592	13.8%	116	16.4%	0.043
Dyslipideamia	4289	10.6%	94	13.3%	0.018
GERD	3524	8.7%	107	15.1%	<0.001
OSAS	3547	8.7%	86	12.1%	0.001
Arthrosis	424	1.0%	9	1.3%	0.550
Comorbidities					
Cardiovascular disease					
MI	695	1.7%	25	3.5%	<0.001
Heart failure	514	1.3%	20	2.8%	<0.001
Vascular disease	91	0.2%	5	0.7%	0.006
CVA	466	1.1%	13	1.8%	0.083
Pulmonary disease	2146	5.3%	43	6.1%	0.346
GI ulcer	286	0.7%	6	0.8%	0.647
Liver disease	92	0.2%	2	0.3%	0.753
Renal disease	55	0.1%	2	0.3%	0.284
Malignancy	56	0.1%	2	0.3%	0.96
Connective tissue disease	40	0.1%	0	0.0%	0.399

*Posthoc testing with Bonferroni correction revealed no statistical differences within groups

*SD*, standard deviation; *ASA*, American Society for Aneasthesiologists; *GERD*, gastro-esophageal reflux disease; *OSAS*, obstructive sleep apnea syndrome; *MI*, myocardial infarction; *CVA*, cerebrovascular accident; *GI ulcer*, gastrointestinal ulcer

A total of 31,452 patients (77.4%) underwent a gastric bypass procedure and 9.188 patients underwent a sleeve gastrectomy (22.6%). An overview of all types of operations and complications is depicted in Table 2. A reoperation within 30 days of index operation was necessary for 709 patients, of which 196 patients (2.4%) were reoperated following sleeve gastrectomy and 513 patients (1.6%) were reoperated following a gastric bypass procedure, *p*=<0.001.

**Table 2:** Overall comparison of operative data for patients who underwent a reoperation following bariatric versus those who did not suffer such a major complication

Parameter	Total population		Reoperation		*p*-value
Total no	40,640		709	1.7%	
Procedure type					
Gastric sleeve	9188	22.6%	196	27.6%	0.001
Gastric bypass	31,452	77.4%	513	72.4%	
Roux and Y	27,526	87.5%	454	88.5%	0.001
Single anastomosis	3051	9.7%	33	6.4%	
Banded bypass	875	2.8%	26	5.1%	
Admission					
Length of stay (mean, IQR)	1	(1 - 2)	4	(1 - 6)	<0.001
Complications					
Perioperative	491	8.7%	0	0%	<0.001*
No readmission	1186	21.0%	435	61%	
Readmission	1025	18.1%	274	39%	
Reoperation within 30 days of index operation			709		
Following gastric bypass			218	2.4%	<0.001
Following sleeve gastrectomy			491	1.6%	
Death	28	0.1%	11	1.6%	<0.001

*Posthoc testing with Bonferroni correction revealed no statistical differences within groups

*IQR*, interquartile range

Bleeding (42%) and leakage or perforation (24.3%) were the most reported indications for reoperations. A full overview of the indications for reoperations and Clavien-Dindo scores can be found in Table 3.

**Table 3:** Details on patients who underwent a reoperation within 30 days of the index operation

Parameter	Reoperation	
	*N* = 709	%
Clavien Dindo		
Type 3b	563	79.4%
Type 4	139	19.6%
>24-hour monitoring	106	15.0%
Single organ failure	22	3.1%
Multi organ failure (MOF)	11	1.6%
Type 5, death	7	1.0%
Failure to rescue in MOF	5	0.7%
Access type		
Laparoscopy	592	83.5%
Conversion	3	0.4%
Primary open	114	16.1%
Indication for reoperation		
Bleeding	298	42.0%
Anastomotic leak	151	21.3%
Intra-abdominal abcess	25	3.5%
Intestinal obstruction	83	11.7%
Perforation	21	3.0%
Sepsis	7	1.0%
Non-surgical complication	46	6.5%
Not registered	78	11.0%

*MOF*, multi organ failure

**Fig. 1:**
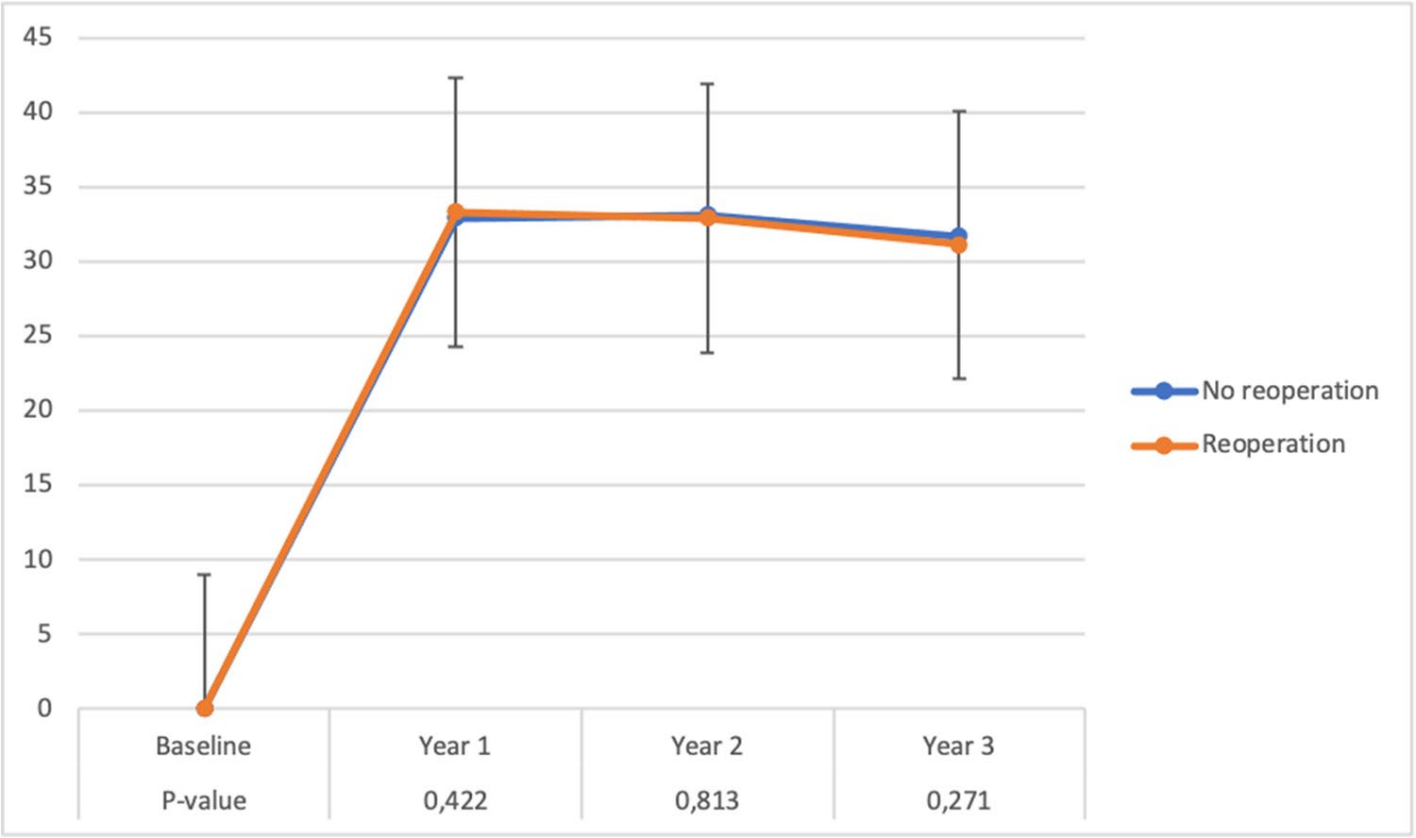
Average total weight loss (TLW) in patients who suffered a major complication requiring reoperating compared to patients without a major complication following bariatric surgery. The error bars represent the standard deviations. *P*-values for the difference between the two groups are depicted below

Follow-up data was collected every year for up to 5 years after surgery; adherence to follow-up was low and decreased over the follow-up period, with 65% complete follow-up at 1 year and 49% complete follow-up at 2 years. Follow-up rates and available data were similar between patients who underwent a reoperation versus those with an uncomplicated postoperative course. As a measurement for case mix, no differences were observed for the type of surgery, complications, and reoperations between the groups during the different follow-up moments. Follow-up data are presented for 1- and 2-year follow-up in Tables 4 and Table 5. At both time points, no differences were observed for patients who were reoperated within 30 days of index operation versus no reoperation for overall weight loss, %TWL and success rate (%TWL > 20%). With %TWL at 12 months being 32.9 ± 8.1 in the overall population, versus 33.3 ± 8.2 in the patients who underwent a reoperation (*p*=0.422). %TWL at 24 months was 33.1 ± 9.2 in the overall population, versus 32.9 ± 8.7 in the patients who underwent a reoperation (*p*=0.813). Alongside, no differences were observed in the resolution of comorbidities at 12 or 24 months. An overview of the overall %TWL over time is presented in Fig. 1. Subgroup analysis, including only patients who underwent a sleeve gastrectomy or only patients who underwent a gastric bypass procedure, revealed no differences in %TWL and no differences in the resolution of comorbidities.

**Table 4:** Follow-up after 12 months following bariatric surgery

Parameter	Total population		Reoperation		*p*-value
Follow-up	26,407	64.9% of the population	429		
Weight loss (mean, SD)	−41.7	14.6	−41.6	13,2	0.874
% TWL (mean, SD)	32.9	8.1	33.3	8,2	0.422
TWL > 20%	25,023	94.8%	405	94.4%	0.754
Remission of comorbidities	Data not available for all patients with comorbidities				
Hypertension	7809	77.5%	147	75.0%	0.39
Diabetes	5075	90.0%	99	90.0%	0.999
Dyslipaedeamia	3636	63.0%	74	67.3%	0.371
OSAS	4381	80.0%	80	77.7%	0.535
GERD	2125	65.4%	48	61.5%	0.631
Arthrosis	7246	71.6%	124	72.9%	0.728
Gastric bypass					
Follow-up	20,046		311		
Weight loss (mean, SD)	42.2	14.3	41.1	12.6	0.909
% TWL (mean, SD)	33.7	7.8	33.9	7.5	0.707
WL > 20%	19,342	96.5%	298	95.8%	0.532
Gastric sleeve					
Follow-up	6361		118		
Weight loss (mean, SD)	40.2	15.2	40.3	14.7	0.909
% TWL (mean, SD)	30.7	8.6	31.9	9.6	0.214
TWL > 20%	5,681	89.3%	107	90.7%	0.763

*TWL*, total weight loss; *OSAS*, obstructive sleep apnea syndrome; *GERD*, gastro-esophageal reflux disease. Remission of comorbidities was defined as either a decreased need (or no need) for therapy of comorbidities, resulting in either decreased intake or dosage of medication, or no longer requiring interventions such as obstructive sleep apnea mask

**Table 5:** Follow-up after 24 months following bariatric surgery

Parameter	Total population		Reoperation		*p*-value
Follow-up	19,726	48.5% of the population	342		
Weight loss (mean, SD)	−41.9	16.2	−41.1	13.2	0.607
% TWL (mean, SD)	33.1	9.2	32.9	8.7	0.813
TWL > 20%	18,175	92.1%	320	93.6%	0.361
Remission of comorbidities	Data not available for all patients with comorbidities				
Hypertension	6439	76.3%	111	66.9%	0.006
Diabetes	4038	87.6%	83	89.2%	0.751
Dyslipaedeamia	3265	66.3%	52	62.7%	0.483
OSAS	3634	83.3%	68	82.0%	0.882
GERD	1759	65.8%	34	59.6%	0.326
Arthrosis	5749	68.9%	97	69.3%	0.999
Gastric bypass					
Follow-up	14,935		237		
Weight loss (mean, SD)	42.8	15.8	41.8	12.2	0.509
% TWL (mean, SD)	34.2	8.8	33.8	7.5	0.652
TWL > 20%	14,134	94.6%	229	96.6%	0.192
Gastric sleeve					
Follow-up	4791		105		
Weight loss (mean, SD)	39.2	17.1	39.5	15.5	0.507
% TWL (mean, SD)	29.9	9.8	30.7	10.6	0.304
TWL > 20%	4041	84.3%	91	86.7%	0.588

*TWL*, total weight loss; *OSAS*, obstructive sleep apnea syndrome; *GERD*, gastro-esophageal reflux disease. Remission of comorbidities was defined as either a decreased need (or no need) for therapy of comorbidities, resulting in either decreased intake or dosage of medication, or no longer requiring interventions such as obstructive sleep apnea mask

A Kaplan-Meier curve revealed no differences in success rate (%TWL>20%) over time between patients who underwent a reoperation within 30 days of index operation versus patients with an uncomplicated course, *p*=0.905. Cox regression was used to correct for baseline characteristics (gender, age, BMI at baseline, comorbidities) and operative data (gastric bypass versus sleeve gastrectomy) and revealed no differences in the success rate over time.

A total of 96 out of 40,640 patients were admitted to the intensive care unit (ICU) and treated for single or multi organ failure. Assessment of follow-up of this subgroup revealed no differences in total weight loss, %TWL, and success rate at 12- and 24-month follow-up.

## Discussion

A major complication, such as a reoperation, within 30 days of a gastric bypass or sleeve gastrectomy does not affect mid-term weight loss or remission of comorbidities during follow-up. Corrections for baseline characteristics and comorbidities further underwrite these findings.

Although rare after bariatric surgery, postoperative complications such as anastomotic leakage or bleeding can have a major impact on these relatively unhealthy patients with the pre-existent comorbid disease, potentially resulting in ICU admission and significantly delayed recovery. Our findings could be of support for those patients that required reoperation, since it confirms that their long-term outcome is as good as those patients that were discharged early without any complications.

Previous studies in gastric cancer patients have shown a worse prognosis in patients who suffered a postoperative complication [16], with major complications leading up to even worse survival outcomes compared to minor complications [17]. Alongside, worse nutritional index values were observed in patients who had postoperative complications following gastrectomy [18]. Further underwriting the importance of assessing long-term weight loss. Similarly to gastrectomy for cancer, baseline characteristics did not affect postoperative nutritional status [19].

Although postoperative weight loss and percentage total weight loss are markers of nutritional status following bariatric surgery, the current study had no information on postoperative micro-nutrient insufficiencies and the effect of complications on micro-nutrients is unclear. Knowledge of micronutrients is important to prevent nutritional complications [20]. Similarly, skeletal muscle mass may be affected by complications, regardless of the measured weight [21].

Although no differences in follow-up outcomes were observed between patients with and without reoperation, in theory, those patients with more severe complications and strongly prolonged hospitalization could still have inferior long-term results compared to those with minor complications. A subgroup analysis of patients treated in the ICU for single or multi organ failure also revealed no differences in weight loss over time. Future analysis of the subgroup of patients that require long-term parenteral nutrition following complications might reveal predictors for worse outcomes in the future.

These results may be taken into account when counseling patients prior to bariatric surgery. It should be noted that reoperations and ICU management can be traumatic for patients and a previous study reported worse quality of life in patients who suffered complications after a gastric bypass procedure measured at 12 and 24 months after surgery [22].

The here presented study is limited by its observational nature and a lack of adequate follow-up data past 2 years follow-up. As obesity surgery is centralized in the Netherlands, patients often return to their own clinics and general practitioners for follow-up and are hence lost to follow-up in the DATO dataset [14]. Analysis of casemix and frequency of complications showed no differences over time, indicating that selection bias is low however may still be present. Although the follow-up data is incomplete, the study still represents an important finding that may help in the counseling of patients prior to bariatric surgery. It should be noted that other major complications, such as repeated stenting and drainages, are not well documented within the DATO dataset, and no conclusions can be drawn on these types of complications.

In summary, major complications requiring reoperations within 30 days of gastric bypass surgery or sleeve gastrectomy did not affect long-term outcomes with regard to weight loss or remission of comorbidities during follow-up. Patients requiring reoperation can be reassured that the long-term results of their bariatric procedure are as good as those patients that were discharged early without any complications. The effects of major complications on micronutrient deficiencies and skeletal muscle mass remain to be determined.
